# Mechanism of eukaryotic origin unwinding is a dual helicase DNA shearing process

**DOI:** 10.1073/pnas.2316466120

**Published:** 2023-12-18

**Authors:** Lance D. Langston, Roxana E. Georgescu, Michael E. O'Donnell

**Affiliations:** ^a^The Rockefeller University, New York City, NY 10065; ^b^HHMI, New York City, NY 10065

**Keywords:** CMG, Mcm10, replication origins, helicase, origin unwinding

## Abstract

DNA replication in all cells begins with the melting of base pairs at the duplex origin to allow access to single-stranded DNA templates which are replicated by DNA polymerases. In bacteria, origin DNA is presumed to be melted by accessory proteins that allow loading of two ring-shaped replicative helicases around single-strand DNA (ssDNA) for bidirectional unwinding and DNA replication. In eukaryotes, by contrast, two replicative CMG (Cdc45-Mcm2-7-GINS) helicases are initially loaded head to head around origin double-strand DNA (dsDNA), and there does not appear to be a separate origin unwinding factor. This led us to investigate whether head-to-head CMGs use their adenosine triphosphate (ATP)-driven motors to initiate duplex DNA unwinding at the origin. Here, we show that CMG tracks on one strand of the duplex while surrounding it, and this feature allows two head-to-head CMGs to unwind dsDNA by using their respective motors to pull on opposite strands of the duplex. We further show that while CMG is capable of limited duplex unwinding on its own, the extent of unwinding is greatly and rapidly stimulated by addition of the multifunctional CMG-binding protein Mcm10 that is critical for productive initiation of DNA replication in vivo. On the basis of these findings, we propose that Mcm10 is a processivity or positioning factor that helps translate the work performed by the dual CMG motors at the origin into productive unwinding that facilitates bidirectional DNA replication.

## Significance

To initiate replication of double-stranded DNA genomes, the two interconnected strands must be separated to allow each strand to serve as a template for DNA synthesis. The initially unwound state is propagated by ring-shaped helicases that surround one strand and move along the DNA backbone to unwind the adjacent DNA for copying by a DNA polymerase. This elongation stage is well characterized in all domains of life, but the initiation of DNA unwinding in eukaryotes is poorly understood. Here we find that two eukaryotic helices, by themselves, provide the motors to unwind the origin by pulling on opposite strands of the DNA backbone while facing one another, thereby breaking the connections between the strands.

The structure of the DNA double helix provides an elegant way to store information, but in order for the information to be read or copied, the hydrogen bonds between the bases of the two strands need to be separated. For transcription, it is sufficient to melt a small bubble of about 10 to 25 base pairs that migrates as the RNA polymerase machinery moves along the DNA, but this approach does not seem to have been adopted for DNA replication in any cellular lifeform despite its simplicity. Replication also begins with melting of a bubble, but the bubble serves as an initiation site for two ring-shaped helicases that facilitate bidirectional replication by surrounding and translocating along one of the two strands and excluding the other strand from traversing the interior of the ring. In bacteria, it is thought that the duplex DNA is melted by a separate initiator protein before the helicase is loaded onto ssDNA. In eukaryotes, it is well established that the helicase is initially assembled around duplex DNA and also transitions to ssDNA translocation, but the nature of the initial duplex unwinding step has remained elusive.

The specification and activation of origins of DNA replication in eukaryotes have been extensively studied in vivo and in vitro, particularly in the yeast systems and in metazoans (1, 2). The process begins with origin “licensing” during the G1 phase of the cell cycle. The Origin Recognition Complex (ORC) is bound to nucleosome-free regions throughout the chromosome that are sequence-specific in budding yeast but not in other well-characterized eukaryotes. Along with Cdc6, origin-bound ORC recruits the heterohexameric Mcm2-7 helicase engine in a complex with Cdt1 to load the ring-shaped Mcm2-7 which then closes to topologically surround duplex DNA. ORC then loads a second Mcm2-7 head to head with the first, and in this double hexameric form, the two helicases are inert and are physically connected across the interface between them (3). Activation of the helicases during the S phase is controlled by a pair of cell cycle kinases and multiple chaperones that convert the two inert Mcm2-7 hexamers at each origin into active helicases through the binding of Cdc45 and the heterotetrameric GINS complex to form CMG (Cdc45-Mcm2-7-GINS) (1, 2).

Most of the steps in this complex process are deeply characterized with a few major exceptions, including the mechanism by which Cdc45 and GINS are assembled by their respective chaperones onto the loaded Mcm2-7 double hexamer to form two 11-subunit CMGs surrounding dsDNA. One of the least understood steps in the process is among the most important: the initial unwinding of origin dsDNA that facilitates the transition of the ring-shaped CMG helicase from a functionally inactive form surrounding duplex DNA at the origin to an active form surrounding ssDNA at the replication fork. The only feature of this process that is well understood is that it does not occur in the absence of Mcm10, a multifunctional DNA-binding protein that also binds to Mcm/CMG (4–9). Mcm10 can bind weakly to Mcm2-7 during the G1 phase, but its binding is greatly enhanced upon addition of Cdc45 and GINS to form CMG in the S phase (10, 11).

Using the reconstituted origin system, the nature of the changes that occur upon addition of Mcm10 was defined as a change from ~0.7 turns of DNA untwisted per CMG in the absence of Mcm10 to ~2 turns of DNA unwound per CMG in the presence of Mcm10, when each CMG surrounds leading strand ssDNA and the lagging strand is excluded from the central channel of the helicase (8). A structural analysis of dual CMG-Pol ε complexes assembled at an origin in the absence of Mcm10 showed only a few base pairs unwound per complex, which is insufficient to facilitate subsequent steps in the initiation process (12). In particular, exclusion of the lagging strand from the central channel of each CMG is critical to origin activation because the two ring-shaped helicases are loaded head to head (N-face to N-face) around dsDNA (13), but unexpectedly they were found to translocate N-face-first on ssDNA at the replication fork (14). Thus, the two CMGs must pass one another to initiate replication, but they can only do so when each CMG surrounds its respective leading strand template ssDNA which requires significantly more unwinding than was observed with CMG alone.

One of the challenges of studying this process using the reconstituted origin system is that all of the proteins necessary for assembling and activating CMG are present in the reaction, so it is difficult to discern the specific role of Mcm10 in unwinding the origin separate from the numerous other proteins that might also participate in this process. To address this challenge, we developed an assay that allows loading of two head-to-head CMGs onto a defined duplex DNA without any other proteins present and showed that CMG unwinds the duplex in the presence of Mcm10 (15). We also developed assays to characterize dsDNA translocation by CMG including the ability to move directionally and to perform work while surrounding duplex DNA. Using these assays, we showed that CMG moves directionally on dsDNA and melts branched base pair arms downstream of the direction of translocation, and these capabilities are also greatly stimulated by Mcm10. Finally, we showed that during duplex DNA translocation, CMG-Mcm10 mainly tracks on one strand of the duplex, the same strand that is used during ssDNA translocation, while the other strand appears to move passively through the central channel via its base-pair connections to the translocation strand.

On the basis of these findings, we proposed that the ATP-driven motors of two head-to-head CMGs at an origin track on opposite DNA strands while surrounding duplex DNA and, because the two rings cannot pass one another, they essentially shear apart the origin dsDNA spanning the two motors (15). As they continue to pull on their respective tracking strands, they eventually produce sufficient ssDNA for exclusion of the lagging strand from the central channel of each CMG. In a separate study, we showed that a similar process occurs during duplex unwinding by two oppositely oriented hexamers of Simian Virus (SV40) T-antigen helicase, suggesting that DNA shearing by head-to-head ring-shaped motors surrounding dsDNA might be a conserved process for initiating origin unwinding in multiple domains of life (16).

To better understand the separate contributions of CMG and Mcm10 to origin unwinding, we investigated multiple aspects of the dsDNA unwinding process, and we show that on its own, CMG is capable of limited unwinding of a 150 bp duplex DNA when it is preloaded onto the substrate using a nonhydrolyzable ATP analog, adenylyl imidodiphosphate (AMP-PNP). We further show that head-to-head CMGs can be loaded onto the model substrate in an unwinding-competent state but they have limited ability to unwind the substrate in the absence of Mcm10. Upon addition of Mcm10, duplex unwinding is greatly stimulated and is completed as quickly as 15 s after addition of Mcm10, suggesting that origin melting occurs extremely rapidly in vivo upon CMG formation as long as Mcm10 is present. These findings lead to the intriguing possibility that Mcm10 might be a limiting factor at origins where CMG is formed but the origin does not fire because, when Mcm10 is present, origin unwinding occurs rapidly.

## Results

### CMG Translocates on One Strand while Surrounding Duplex DNA.

**Fig. 1.**
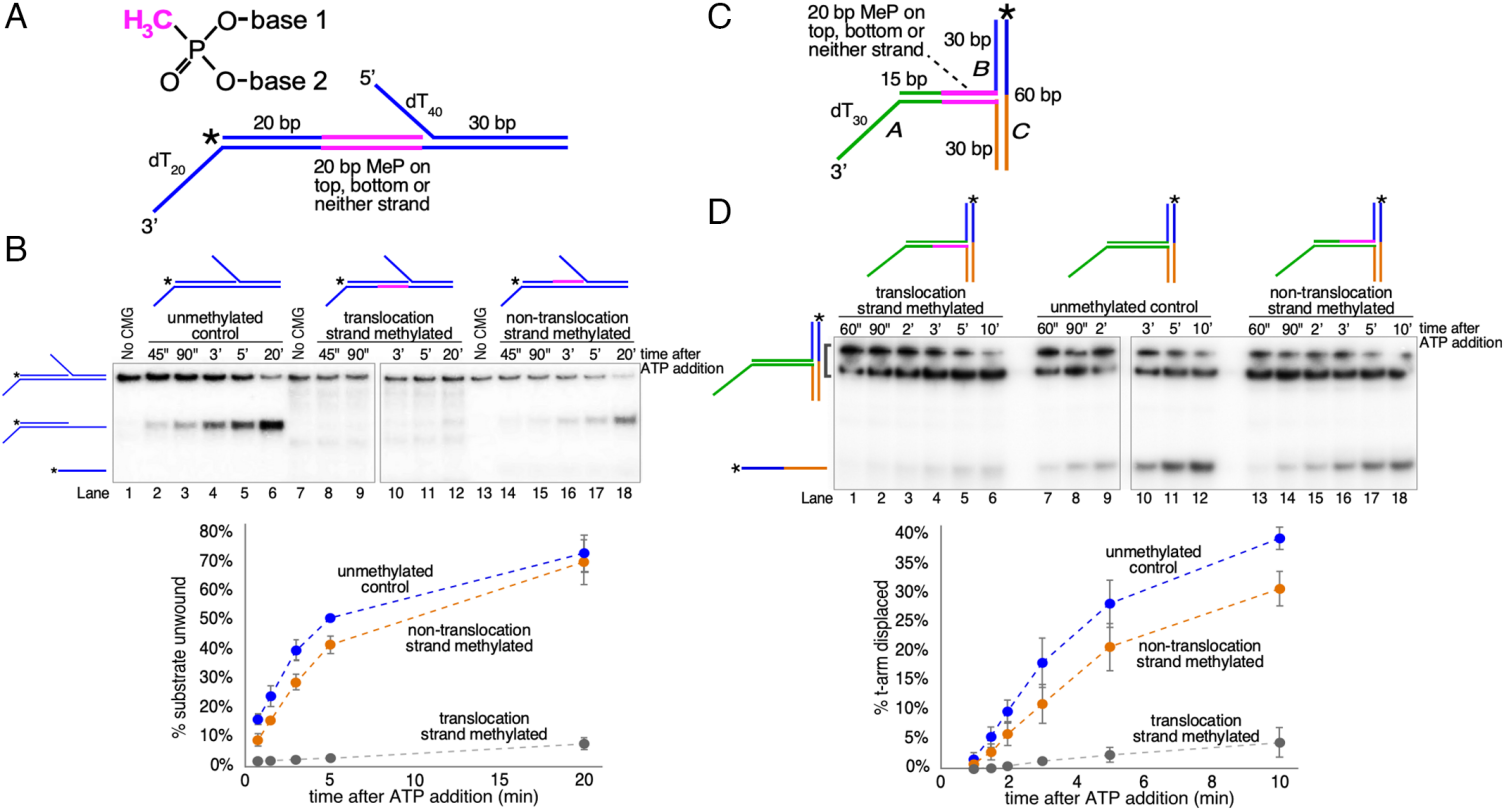
CMG tracks on the leading strand during duplex translocation. Unwinding of two different substrates with 20 nt MeP tracts on the leading (translocation) or lagging (nontranslocation) strand backbone was compared to the unmodified substrate. (*A*) Schematic of the substrate used in panel *B*. A stretch of bases linked by MeP is indicated in pink on the schematic and in the diagrams above the gels in (*B*). The radiolabeled oligo in the substrate is indicated by an asterisk at the 5′ end. At the *Top Left* is a diagram of the MeP linkage showing the charge neutralization on the backbone in pink. (*B*) CMG (40 nM) is preincubated with the substrate (0.5 nM) for 10 min in the presence of 0.2 mM AMP-PNP before starting the reaction by addition of 5 mM ATP along with trap oligos to prevent reannealing of either the flush or 5′-tailed duplex oligo after unwinding (see *Materials and Methods* and *SI Appendix* for details). As shown in pink on the schematics above the gel, the substrate contains 20 charge-neutralizing MeP linkages on the phosphate backbone of the tracking strand, (lanes 7 to 12), the nontracking strand (lanes 13 to 18), or neither strand (lanes 1 to 6). A time course of unwinding is shown in the gels, and the positions of potential products are shown to the *Left* of the gels. The plot at the *Bottom* shows the time course of unwinding of the 5′-tailed duplex with no MeP (blue circles) or 20 MeP linkages on the tracking (gray circles) or nontracking (orange circles) strand. Values in the plots are the average of three independent experiments, and the error bars show the SD. (*C*) Schematic of the T-substrate used in panel (*D*), also showing a stretch of bases linked by MeP in pink. CMG has been shown to load onto the substrate via the 3′ ssDNA dT tail on oligo *A* and melt the cross-bar oligo *C* off the nonhomologous arms of Oligos *A/B* (15). (*D*) Reaction conditions are the same as in (*B*) except that Mcm10 (40 nM) is also added with CMG (20 nM) during the preincubation with the T-substrate and the trap oligo is an excess of unlabeled oligo (*C*). The plot at the *Bottom* shows the time course of unwinding of the 5′-tailed duplex with no MeP (blue circles) or 20 MeP linkages on the tracking (gray circles) or nontracking (orange circles) strand. Values in the plots are the average of three independent experiments, and the error bars show the SD.

We previously showed that on its own, budding yeast CMG can translocate over duplex DNA without unwinding it and can unwind a 5′-tailed duplex tract downstream of the direction of translocation (17). To test whether CMG tracks on one or both strands of the duplex during active translocation, we used the substrate shown in Fig. 1*A* and modified 20 nt of either the leading or lagging strand of the flush (untailed) duplex tract to eliminate the charge on the DNA backbone by converting the phosphodiester linkages to methylphosphonate (MeP) (15, 18).

As shown in Fig. 1*B*, when the MeP_20_ stretch is on the lagging (nontracking) strand (lanes 13 to 18), the downstream tailed oligo is unwound to the same extent as in the unmodified substrate (lanes 1 to 6). By contrast, when the MeP_20_ stretch is on the leading (tracking) strand (lanes 7 to 12), unwinding of the downstream tailed oligo is almost completely eliminated, indicating that CMG tracks on the same strand during duplex translocation as during single-strand DNA translocation. We previously showed that this is a property of CMG-Mcm10 (15), but the results in Fig. 1 confirm that CMG tracks on the leading strand of the duplex whether Mcm10 is present or not. This result is also consistent with two recent cryo-EM structures of CMG on duplex DNA that showed that contacts with the leading strand template backbone are similar to those between CMG and ssDNA (12, 19). Together, these results suggest that the mechanism of CMG translocation is similar regardless of whether CMG is surrounding ssDNA or dsDNA and that contacts between CMG and the lagging strand template in dsDNA are negligible.

### CMG-Mcm10 Duplex Translocation is Greatly Impaired by Neutralizing Charge on the Tracking Strand Backbone.

The effect of the leading strand MeP_20_ tract on unwinding of the substrate in Fig. 1*B* (reduced by almost 10-fold) is much stronger than our previously published results with CMG-Mcm10, where a 10 nt MeP stretch on the leading strand only reduced unwinding of a fully duplex T-substrate by about threefold (15). To determine the effect of a longer MeP_20_ stretch on unwinding by CMG-Mcm10, we used the substrate from our previous work but expanded the MeP tract from 10 nt to 20 nt. This substrate, made from three oligos, has a 3′ dT_30_ tail for loading CMG onto the duplex and two 30 bp arms that are noncomplementary so they cannot branch migrate (Fig. 1*C*). Thus, in order for the third oligo (oligo C in Fig. 1*C*) to be unwound, CMG-Mcm10 must exert sufficient force to break base pairs ahead of the direction of translocation while surrounding and translocating over dsDNA.

Using this substrate (Fig. 1*D*), when the MeP_20_ stretch is on the nontracking strand (lanes 13 to 18), the third (C) oligo is unwound to about the same extent as in the unmodified substrate (lanes 7 to 12), but when the MeP_20_ stretch is on the tracking strand (lanes 1 to 6), unwinding is reduced by ninefold at the 10-min time point compared to the unmethylated control. The reduction in unwinding in this assay is similar to that in Fig. 1*B*, suggesting that tracking on the leading strand while surrounding duplex is a property of CMG whether Mcm10 is present or not. Together with our previous work, the evidence that CMG and SV40 T-Antigen both track on the same strand of DNA while surrounding ssDNA or dsDNA suggests that this is a property of many ring-shaped helicases, not just those that are dedicated dsDNA translocases (15, 16).

### Unwinding of Duplex DNA by Oppositely Facing CMGs.

**Fig. 2.**
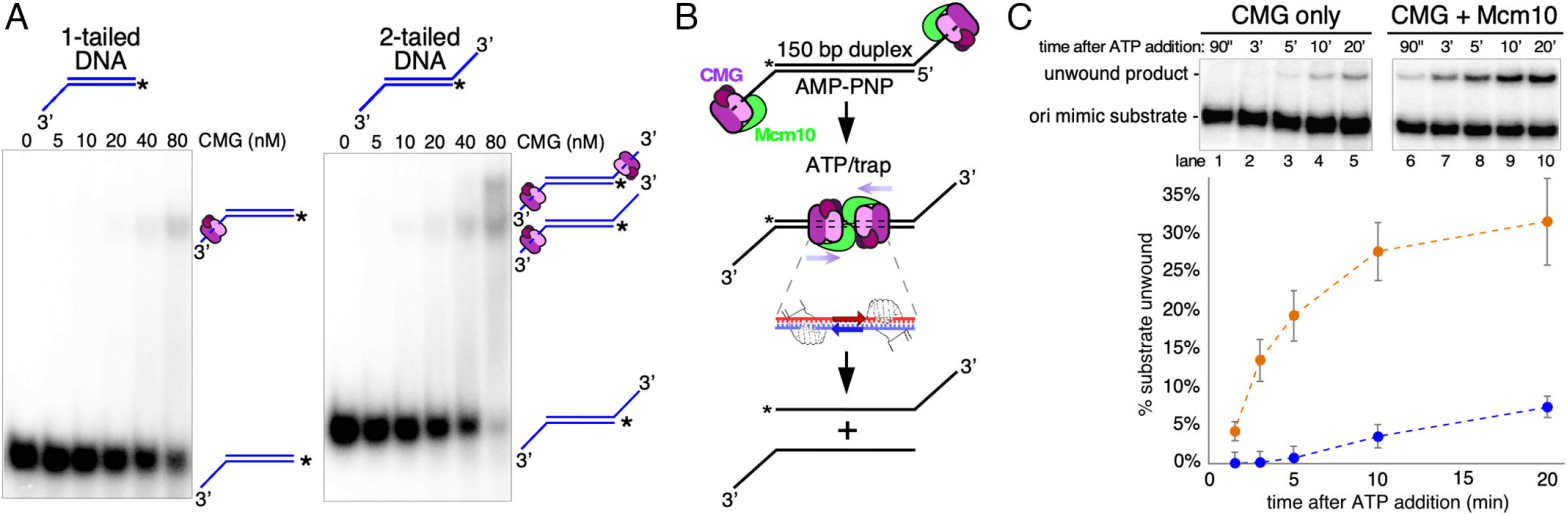
Preloading CMG-Mcm10 on DNA with AMP-PNP gives robust unwinding of the origin mimic DNA. (*A*) Electrophoretic mobility shift assay (EMSA) assays were performed using AMP-PNP for loading CMG onto 150 bp duplexes containing either one or two 3′ single-strand dT tails (20). CMG was titrated into reactions containing DNA and 0.2 mM AMP-PNP and then analysed for gel shifts by neutral PAGE. (*Left*) The DNA containing only one 3′ tail gave only one gel shift band. (*Right*) The DNA containing two 3′ tails gave two gel shift bands. The presumed interpretation of the bands is shown to the *Right* of the gels. EMSA assay reactions were repeated twice with the same result. (*B*) Scheme of duplex unwinding by CMG ± Mcm10. Preincubation with AMP-PNP allows binding of one CMG to each tail of the substrate (*Top*). Upon addition of ATP, the two CMGs translocate over duplex DNA (*Middle*) and shear apart the DNA with each motor acting on different strands of the duplex (hands in *Inset*) with the right hand pulling on the top strand (red) and the left hand pulling on the bottom strand (blue), leaving the two strands fully separated (*Bottom*). (*C*) CMG (40 nM) ± Mcm10 (80 nM) is preincubated with the substrate (0.5 nM) for 10 min in the presence of 0.2 mM AMP-PNP before starting the reaction by addition of 5 mM ATP and an ssDNA trap oligo that quenches further CMG loading (15) and also anneals to the unwound radiolabeled product, creating a forked structure that migrates at a distinct position in the gel from the substrate as indicated to the *Left* of the gels. The native PAGE gels at the *Top* show a time course of results using CMG only (lanes 1 to 5) or CMG + Mcm10 (lanes 6 to 10). The plot at the *Bottom* shows unwinding of the substrate as the averages of three independent trials for CMG only (blue circles) or CMG + Mcm10 (orange circles). The error bars show the SD.

The fact that CMG tracks on one strand of the duplex while surrounding both strands fulfills one of the key criteria for initiating duplex unwinding at an origin, but in our previous experiments, we saw no evidence that head-to-head CMGs loaded onto dsDNA could unwind a 150 bp duplex in the absence of Mcm10 (15). The substrate in these assays was a linear 150 bp ARS1 duplex with 3′-ended poly-dT loading sites for CMG at both ends to simulate an origin in which two CMGs are oriented head to head around dsDNA (Fig. 2*B*). Because Mcm10 might help to load CMG onto the substrate in these experiments, it was not possible to separate out the role of Mcm10 in unwinding the duplex from its potential role in helping to load CMG.

To focus more specifically on the separate roles of CMG and Mcm10 in duplex unwinding, we sought to establish conditions to promote efficient substrate loading of CMG in the absence of Mcm10 (20). We have previously shown that CMG binds efficiently to the 3′ end of ssDNA in the presence of AMP-PNP (14), so we first tested the binding of CMG to the origin duplex substrate by gel-mobility shift (Fig. 2*A*). Using a control substrate with only one 3′ poly-dT loading tail, we observed a single gel-shifted band after incubation with CMG and AMP-PNP (Fig. 2 *A*, *Left* gel). By contrast, the assay substrate with 3′ tails at both ends showed two distinct gel mobility shifts as the CMG concentration was increased (Fig. 2 *A*, *Right* gel). These results not only confirm that the preloading step promotes binding of CMG to the substrate, but they also confirm that only one CMG loads onto each tail with AMP-PNP, so when a suitable trap is added upon initiating the reaction with ATP, any subsequent unwinding is attributable to a single pair of head-to-head CMGs meeting on the duplex DNA.

Guided by the mobility shift data, we preincubated CMG with the two-tailed 150 bp duplex substrate for 10′ with AMP-PNP and then started the unwinding reaction by addition of ATP along with a trap oligo that binds to the unwound radiolabeled strand and shifts its mobility in the gel (scheme in Fig. 2*B*). The trap also serves to prevent further loading of CMG onto the substrate as demonstrated previously (15). As shown in Fig. 2*C*, CMG alone exhibits very limited unwinding of the substrate over time (Fig. 2*C*, lanes 1 to 5) and the reaction is greatly stimulated by addition of Mcm10 (Fig. 2*C*, lanes 6 to 10), as previously observed, particularly at earlier time points (unwinding is stimulated >20-fold at the 5′ time point, e.g.). Although the CMG-only activity is weak, this is a demonstration of duplex unwinding by head-to-head CMGs in the absence of Mcm10, indicating that CMG is capable of exerting sufficient force to facilitate the origin unwinding reaction on its own under ideal conditions, though this has not been observed in vivo (4–6, 21). Control reactions showing that Mcm10 does not unwind these substrates without CMG were published previously (15).

**Fig. 3.**
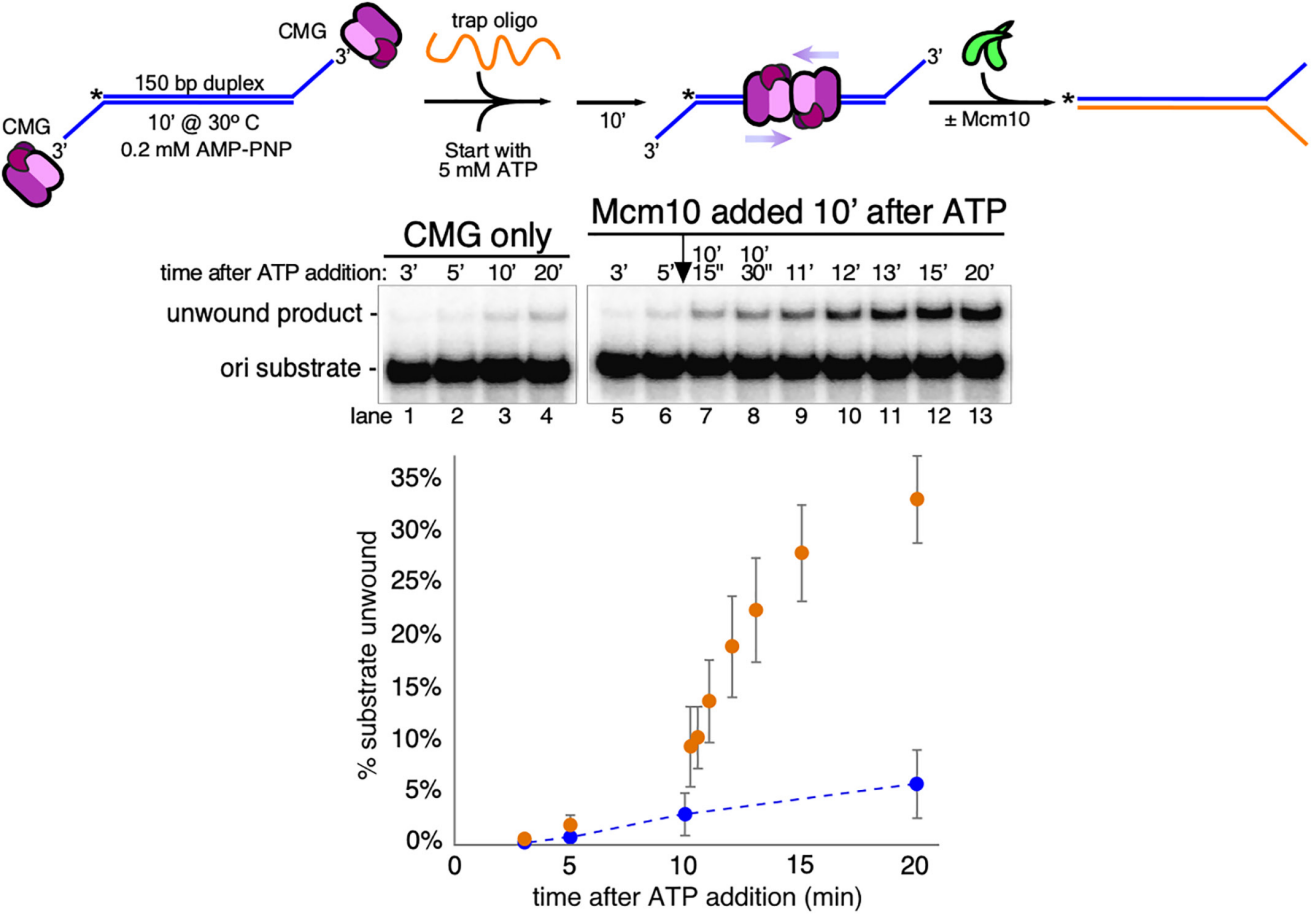
Mcm10 promotes rapid and efficient unwinding of origin mimic substrate by preloaded CMG. The reaction in Fig. 2*C* was repeated, but, as shown in the scheme at the *Top*, CMG (40 nM) was allowed to translocate onto the duplex substrate for 10′ in the presence of ATP before addition of Mcm10 (120 nM). The gels show unwinding by CMG only with no addition of Mcm10 as a control (lanes 1 to 4) or addition of Mcm10 10′ after starting the reaction with ATP (lanes 5 to 6 are prior to the addition of Mcm10; lanes 7 to 13 are after addition of Mcm10). The plot at the *Bottom* shows unwinding by CMG only (blue circles) or by CMG with Mcm10 added 10′ after ATP (orange circles). Values in the plots are the average of three independent experiments, and the error bars show the SD.

The finding that CMG unwinds the substrate very slowly and to a limited extent suggested the possibility that two CMGs are frequently loaded head to head on the substrate in this reaction but in most cases the opposing motors are not sufficiently processive to completely unwind the 150 bp duplex. To test this idea and to further examine the stimulation of unwinding by Mcm10, we preincubated CMG with AMP-PNP and the substrate as above and then added ATP and trap for a further 10′ before addition of Mcm10 (Fig. 3). As before, CMG slowly unwound the substrate up to the 10′ time point and beyond in control reactions without addition of Mcm10 (Fig. 3, lanes 1 to 4 and 5 to 6). Upon addition of Mcm10, however, unwinding was greatly stimulated within 15 to 60 s, suggesting that binding of Mcm10 to CMG is very rapid and can facilitate an almost instantaneous increase in the efficiency of the unwinding reaction (Fig. 3, lanes 7 to 13). Possible explanations for how this might occur will be discussed below. This result also confirms that head-to-head CMG complexes were preloaded onto the assay substrate prior to the addition of Mcm10 but were, for the most part, unable to efficiently unwind the duplex without Mcm10.

One simple explanation for how Mcm10 promotes duplex unwinding by CMG is that it uses its internally located DNA-binding domain to bind the unwound ssDNA, thereby preventing backtracking by CMG as previously observed during ssDNA translocation and fork unwinding (22, 23). Indeed, backtracking is commonly observed with replicative helicases when unwinding is not accompanied by DNA polymerization (24, 25). To examine this possibility, we tested the ability of other well-characterized DNA-binding proteins to promote duplex unwinding using the assay from Fig. 2*B*, but we did not observe any stimulation using either yeast RPA or *Escherichia coli* single-strand binding protein (SSB) (*SI Appendix*, Fig. S1). We also tested an N-terminally truncated Mcm10 protein that retains the internal DNA-binding domain, but this protein did not stimulate duplex unwinding. (*SI Appendix*, Fig. S2). Together, these results indicate that the ability of an accessory protein to bind ssDNA is not, on its own, sufficient to promote duplex unwinding by CMG and further suggest that Mcm10 has additional features that contribute to its function at origins of replication, including its ability to bind to CMG and to form multimers across the N-faces of two oppositely oriented CMGs (9–11, 26–28).

### Effects of DNA Sequence on Unwinding by CMG-Mcm10.

**Fig. 4.**
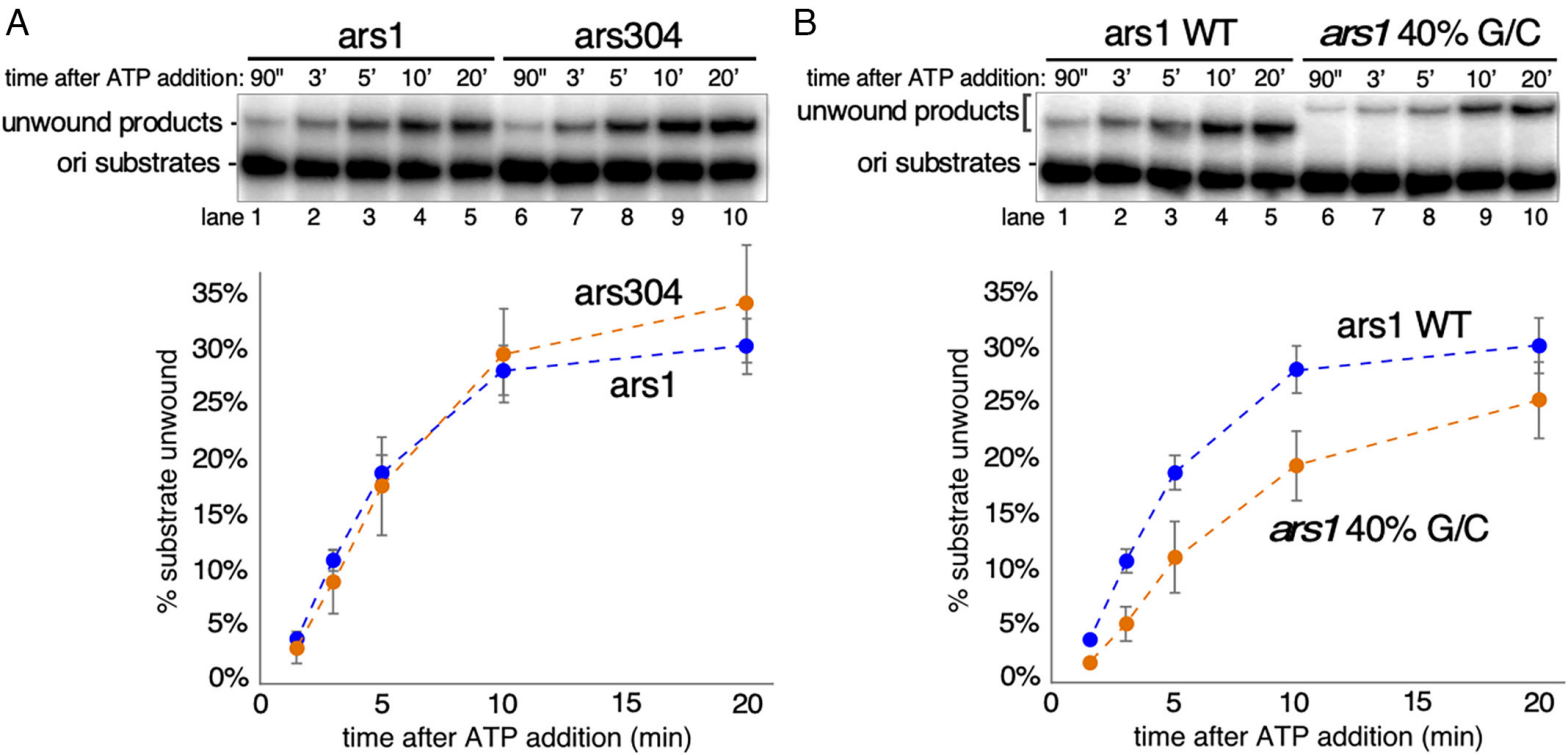
Sequence dependence of duplex unwinding by CMG-Mcm10. The reaction in Fig. 2*C* was repeated with different substrates to show the effect of DNA sequence on unwinding by CMG-Mcm10. (*A*) CMG-Mcm10 unwinding of ars1 (lanes 1 to 5), the sequence used in Fig. 2*C*, is compared with ars304 (lanes 6 to 10), a weak origin in vivo. (*B*) The WT ars1 sequence (29% G/C content, lanes 1 to 5) is compared to a modified version of ars1 with a 40% G/C content (lanes 6 to 10).

The assay we have used to show origin DNA unwinding by CMG-Mcm10 is based on the 150 bp duplex sequence surrounding the well-characterized and highly active ARS1 (systematic name ARS416) origin of budding yeast (29). To investigate whether the results we have observed depend on the DNA sequence used, we repeated the experiment of Fig. 2 using another origin sequence, ARS304, that shows very weak origin activity in vivo despite being an apparent site of Mcm loading (30, 31). As shown in Fig. 4*A*, unwinding of the ARS304 sequence was indistinguishable from that of the ARS1 sequence. These results not only confirm the reproducibility of the data from ARS1, they also provide preliminary evidence that the ability of a particular origin sequence to be unwound by CMG-Mcm10 does not contribute to the frequency with which that origin fires in vivo.

When analyzing the sequences used for ARS1 and ARS304 in Fig. 4*A*, we noticed that both 150-mers had the exact same A/T content, 71.3%, consistent with the fact that origin sequences in yeast are A/T-rich compared to the genome as a whole (~62%) (32). To further examine the influence of DNA sequence on duplex unwinding by CMG-Mcm10, we modified the sequence of the ARS1 150-mer duplex to decrease the A/T content to 60% (*SI Appendix*, Fig. S3), while maintaining the ACS sequence contained in most origins. We reasoned that one explanation for the general A/T-richness of origin sequences in yeast is that A/T base pairs are less stable than G/C base pairs so A/T-rich sequences would be easier to unwind. Indeed, when we examined CMG-Mcm10 unwinding of the altered 40% G/C ARS1 derivative (Fig. 4*B*), we saw a reduction in unwinding compared to the unmodified ARS1 sequence, especially in the earlier time points (≤5 min).

## Discussion

### Head-to-Head Motors Positioned for Duplex DNA Unwinding.

**Fig. 5.**
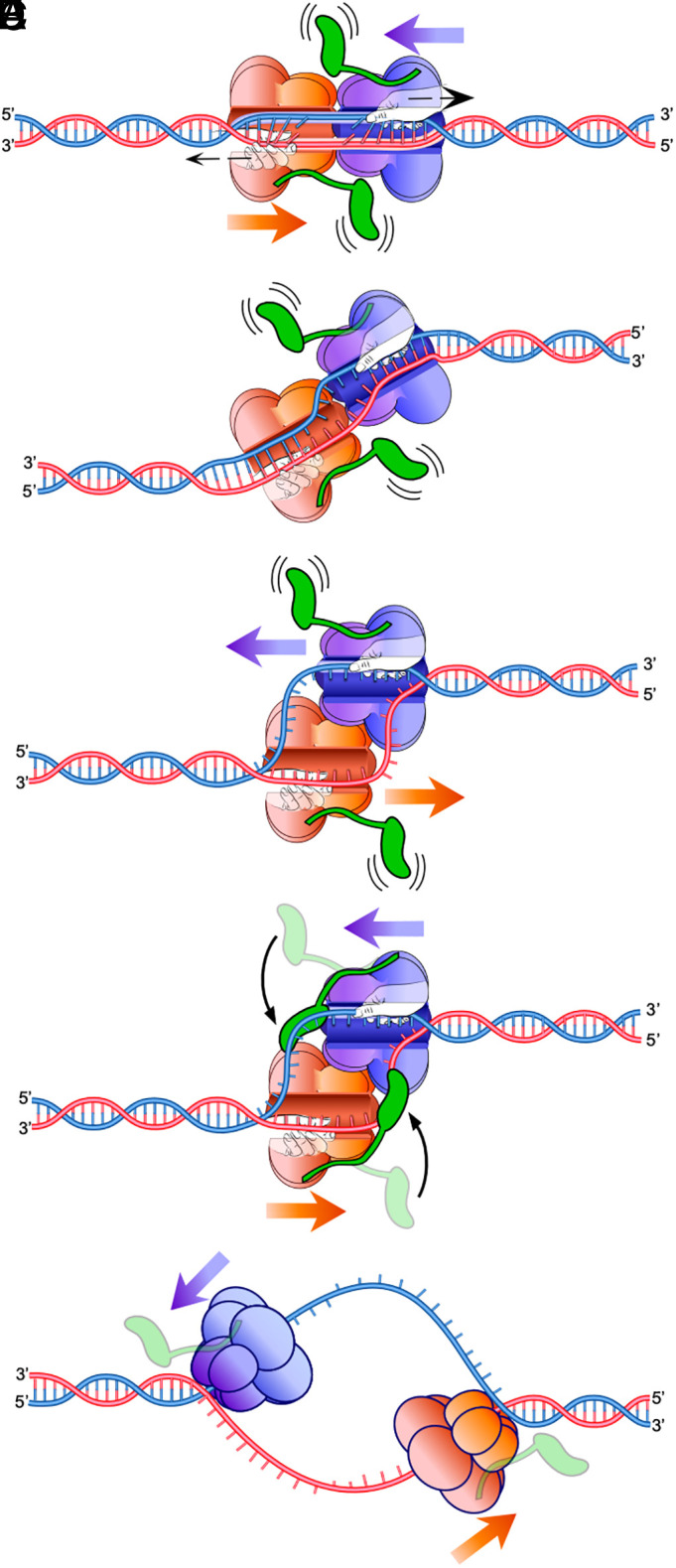
Conceptual model of origin unwinding by CMG-Mcm10. The data presented here suggest a model for origin unwinding by head-to-head CMGs in the presence of Mcm10. (*A*) Head-to-head CMGs (orange and purple) cannot pass one another while surrounding dsDNA so instead the motors (depicted as hands pulling on the DNA) translocate their respective tracking strands of DNA through the central channel without moving forward, breaking the intervening DNA base pairs. In this panel, Mcm10 is depicted in green as binding to the side of CMG (9) with the DNA-binding domain disengaged from DNA. (*B*) As the motors continue to track on their respective strands and (*C*) more origin DNA becomes single-stranded, the DNA-binding domain of Mcm10 engages the DNA, (*D*) thereby providing an additional grip on the DNA that may prevent backsliding by CMG. In addition to binding the DNA, the Mcm10 molecules bound to each CMG may dimerize across the interface between the two helicases, providing additional connections to promote productive unwinding. CMG-Mcm10 also begins to draw the tracking strand out of the central channel of the opposite CMG through dynamic interfaces that spontaneously open and close (see text for references). As this process continues, the tracking strand of each CMG is fully extruded from the central channel of the opposing CMG, and (*E*) the two helicases pass one another to initiate bidirectional replication.

The recent finding that CMG helicase translocates on ssDNA with the C-tier motors at the back pushing the N-tier ring ahead created both a dilemma and a potential explanation for the initial mechanism of unwinding at eukaryotic origins of replication (14). The dilemma stems from the fact that the two CMGs at each origin are formed around dsDNA with their N-tiers facing one another. Thus, in order for each CMG to leave the origin and initiate replication fork unwinding, the two CMGs must first pass each other while surrounding ssDNA, but how the transition from surrounding dsDNA to translocating along ssDNA occurs was unknown. At the same time, the opposing orientation of the two CMG motors on dsDNA provided a simple and straightforward explanation for how origin DNA is initially unwound (15). By pulling on opposite strands while surrounding duplex DNA, the two CMG motors can essentially shear apart the DNA between them, providing sufficient unwound DNA for each CMG to exclude one strand from the central channel and translocate past each other to initiate bidirectional DNA replication (Fig. 5). In vivo and in reconstituted origin replication reactions, Mcm10 is absolutely required for replication initiation under normal circumstances (4–8).

### Mcm10 as a Processivity Factor for CMG.

The present work focuses on the separate functions of CMG and Mcm10 in the unwinding reaction, and we show that even in the absence of Mcm10, CMG tracks on only one of the two strands (the eventual leading strand template) while surrounding and translocating along duplex DNA (Fig. 1*A*). Accordingly, two CMGs loaded head to head around a long duplex template are capable of limited unwinding by tracking on opposing leading strand templates and shearing apart the duplex (Fig. 2). The efficiency of unwinding is strongly and rapidly stimulated by addition of Mcm10, and no other DNA-binding protein can substitute for Mcm10 in this reaction (Figs. 2 and 3 and *SI Appendix*, Fig. S1).

We previously showed that Mcm10 also greatly stimulates unwinding of Y-shaped fork structures by CMG in vitro and mildly stimulates the rate of fork progression in reconstituted replication reactions, which has also been demonstrated by others (28, 33). In single-molecule studies of fork unwinding, *Drosophila* CMG exhibited limited processivity and frequently paused and backtracked on the DNA, which appears to be a common feature of ring-shaped replicative helicases (22, 34). Backtracking at a functional replication fork is likely prevented by the presence of a DNA polymerase filling in the leading strand template as in the bacterial and phage system (35–37), but at the replication origin, we propose that Mcm10 fulfills this role.

Mcm10 binds to DNA, and it binds extensively to the N-face of CMG where it is perfectly positioned for access to newly unwound DNA produced by the action of either the same or the opposed CMG motor (9–11, 28). We previously showed that Mcm10 enables CMG to bypass stringent blocks on the lagging strand that slow the progress of CMG fork unwinding, suggesting that Mcm10 binding to the front face of CMG may alter the way it interacts with the DNA and this capability may also contribute to its role in fork unwinding (33). A strong indication of the additional processivity imparted to CMG by Mcm10 is the fact that, when Mcm10 is present, CMG is able to displace streptavidin bound to two closely spaced biotins on the leading strand, a block that completely prevents translocation by CMG alone (17, 33). Structural and biochemical data from maize (*Zea mays*) and *Xenopus* indicate that the minimal ssDNA-binding site of the Mcm10 OB/ZnF domain is ~8 to 10 nucleotides (38, 39), so this would appear to be the minimum amount of unwinding necessary to promote Mcm10 engagement with the unwound DNA.

In addition to promoting forward motion of the CMG motors via DNA binding, Mcm10 may also increase the efficiency of origin unwinding by its ability to form homodimers and higher-order multimers across the face of the two opposing CMGs (40). In this way, Mcm10 may effectively dimerize the two CMGs, thereby limiting their ability to slip backward or promoting an orientation of the two CMGs that helps reduce the force needed to shear the DNA. This property may also help explain the unique ability of Mcm10 to promote the unwinding reaction as compared to other DNA-binding proteins (*SI Appendix*, Fig. S1). Yeast Mcm10 was previously shown to form trimers upon binding to ssDNA (27), so binding to the newly unwound origin DNA may also promote multimerization of Mcm10. While we have focused on the roles of Mcm10 binding to CMG, to DNA, and to itself in promoting origin unwinding, there are additional ways in which Mcm10 might promote origin unwinding. For example, it was previously shown that Mcm10 modestly stimulates the ATPase activity of Mcm2-7, and ATP hydrolysis by CMG is required for stimulation of origin initiation (8). Thus, Mcm10 might also contribute to origin unwinding by stimulating the ATPase activity of CMG or by promoting particular allosteric changes in CMG that enhance the force of DNA translocation.

### Mechanism of Duplex Unwinding By Head-to-Head Motors.

We have now established three principles that define a mechanism for unwinding of duplex DNA at origins of replication by head-to-head helicases in eukaryotes and some of their DNA viruses (15, 16). First, the direction of ssDNA translocation (N-face first for CMG) implies that the two head-to-head helicases must pass one another rather than moving away from one another at the origin (14, 16). Second, the ring-shaped helicases track on only one of the two DNA strands while surrounding duplex DNA (Fig. 1). Third, the helicases generate sufficient unwinding force to break base pairs while translocating on dsDNA (Figs. 1–4). Together, the work performed by the motors of two head-to-head helicases surrounding dsDNA and acting on opposite strands of the duplex is sufficient to melt long stretches of dsDNA (Figs. 2–4). In eukaryotes, the ability to bring about efficient origin melting is strongly dependent on Mcm10 as shown in Figs. 2 and 3.

On the basis of these features of CMG as demonstrated here and previously, we propose a refined model for unwinding of the origin DNA (Fig. 5). The process begins with two CMG helicase motors starting to track on their respective single strands while surrounding duplex DNA (Fig. 5*A*). Although Mcm10 binds at the N-face of CMG (9, 28), the exact location of Mcm10 on CMG prior to origin unwinding is still to be determined. There is strong evidence that Mcm10 is bound to Mcm2-7 in G1-phase but becomes more tightly bound upon CMG formation, consistent with Mcm10 binding to Cdc45 and the Psf1/2 subunits of GINS (9–11, 41). The data from Fig. 3 indicate that Mcm10 binds to CMG rapidly in a way that greatly enhances the efficiency of origin unwinding, so it is likely that unwinding occurs immediately upon CMG formation in vivo, at least when Mcm10 is present. As the opposing CMGs continue tracking, ssDNA becomes exposed (Fig. 5 *B* and *C*) and provides a potential binding site for the Mcm10 DNA-binding domain (Fig. 5*D*). Binding of Mcm10 to ssDNA ahead of the direction of CMG translocation could promote extraction of the nontracking strand from the central channel of the opposite CMG through dynamic, flexible interfaces in CMG that have been extensively documented (see below). Finally, concomitant with extraction of the nontracking strand from their central channels, the two CMGs pass one another to initiate bidirectional DNA replication at diverging forks (Fig. 5*E*).

The evidence that ring-shaped helicases are dynamic, not static, structures is abundant not just for CMG but for virtually every replicative helicase studied to date. Functional forms of *E. coli* DnaB, SV40 T-Antigen, and CMG have all been shown to be closed rings by structural analysis but also to self-load onto circular DNA in vitro, which requires spontaneous opening of the interface between adjacent protomers within the ring (42–46). Both phage T7 helicase and CMG have been shown to switch between ssDNA and dsDNA translocation at a replication fork, which also requires spontaneous ring opening (47, 48). Thus, once the duplex DNA at an origin is unwound by CMG-Mcm10, we propose that passage of the nontracking strand to the outside of CMG can easily be accommodated by the dynamic nature of the interfaces between adjacent Mcm subunits. Although such a process bears the risk of CMG falling off the DNA, the motors remain bound to the tracking strand throughout and Mcm10 may provide an additional grip to DNA through its attachment to CMG at the origin.

## Materials and Methods

### Reagents and Proteins.

Radioactive nucleotides were from Perkin Elmer, and unlabeled nucleotides were from GE Healthcare. DNA modification enzymes were from New England Biolabs. CMG and Mcm10 were overexpressed and purified as previously described (33, 49, 50). Protein concentrations were determined using the Bio-Rad Bradford Protein stain using BSA as a standard. DNA oligonucleotides were from Integrated DNA Technologies except for those with Mep linkages which were from Biosynthesis or Gene Link.

### DNA Unwinding Assays.

Helicase assays made use of synthetic oligonucleotides configured as illustrated in the figures of this report. Further details are available in *SI Appendix*. Reactions were performed at 30 °C and typically contained 40 nM CMG and, where indicated, 80 nM Mcm10 along with 0.5 nM radiolabeled DNA substrate in 20 mM Tris Acetate pH 7.6, 5 mM DTT, 0.1 mM ethylenediaminetetraacetic acid (EDTA), 10 mM MgSO_4_, 50 mM KCl, and 40 μg/mL BSA in a final volume of 55 μL. Final concentrations of CMG and Mcm10 are indicated in the figure legends. CMG ± Mcm10 was preincubated at 30 °C with the DNA for 10 min in the presence of 0.2 mM AMP-PNP, and reactions were started by addition of 5 mM ATP along with 20 nM unlabeled trap DNA to prevent reannealing of unwound product.

### EMSA Assays.

Binding reactions were performed by incubating 0.5 nM ^32^P-DNA with increasing amounts of CMG (as indicated) in a 10 μL reaction containing 20 mM Tris-acetate, 8% glycerol, 0.02 mM EDTA, 10 mM Na-acetate, 10 mM MgSO_4_, and 0.2 mM AMP-PNP. Reactions were incubated for 60 min at 30 °C. Further details are available in *SI Appendix*.

## Supplementary Material

Appendix 01 (PDF)

## Data Availability

All study data are included in the article and/or *SI Appendix*.
