# Supplementary material for: Mechanism of eukaryotic origin unwinding is a dual helicase DNA shearing process
Source: Proc Natl Acad Sci U S A. 2023 Dec 18;120(52):e2316466120. doi: 10.1073/pnas.2316466120 (PMC10756200; doi:10.1073/pnas.2316466120)
Supplement: Supplementary file 1 — Appendix 01 (PDF) [file pnas.2316466120.sapp.pdf]

Supplementary Information Appendix for:

Mechanism of eukaryotic origin unwinding is a dual helicase DNA shearing process

Lance D. Langston[1,2] , Roxana E. Georgescu[1,2] and Mike E. O'Donnell[1,2,*]

1. The Rockefeller University
2. Howard Hughes Medical Institute
1230 York Avenue
New York City, New York 10065

This Appendix includes:
Detailed Experimental Procedures
3 Supplementary Figures
1 Table
References for Supplementary Material

Detailed Experimental Procedures

Additional Proteins

Yeast RPA and E. coli SSB used in ***SI Appendix* Figure S1** were overexpressed in E. coli and purified as previously described for RPA (1) and SSB (2). Mcm10$^{129\text{-}571}$ (Mcm10ΔN) used in ***SI Appendix* Figure S2** was overexpressed in E. coli with an N-terminal His_6 tag and a C-terminal 3X FLAG tag. Cells were grown to OD_{600}=0.6 at 30°C and induced with 0.5 mM IPTG. Cells were harvested after 2 hours by centrifugation at 5000 rpm in a Thermo Fisher RC3BP centrifuge and H6000A rotor for 30 min at 4 °C. Cells were resuspended in Nickel Buffer (50 mM Tris pH 8.0, 10% Sucrose, 500 mM NaCl and 1 mM EDTA) plus *E. coli* protease inhibitors (Sigma). Cells were lysed using a continuous flow pressure cell (Avestin C50) at 4 °C and cell debris was removed by centrifugation in a Thermo Fisher Lynx 6000 Rotor F14-6-250 at 12,500 rpm for 1 hour at 4 °C. The supernatant was loaded onto a 1 ml HisTrap HP column (GE Healthcare) that was pre-equilibrated with Nickel Buffer and then eluted with FLAG Buffer (50 mM Tris pH 8.0, 10% glycerol, 500 mM NaCl and 1 mM EDTA) supplemented with 70 mM imidazole. Peak fractions were pooled and loaded onto 600 μl ANTI-FLAG M2 Affinity Gel (Sigma) that was pre-equilibrated in FLAG buffer. After loading, the column was washed with 12 ml FLAG buffer and then eluted with FLAG buffer supplemented with 0.15 mg/ml 3X FLAG peptide (EZBiolab, Carmel, IN).

DNA substrates. For all radiolabeled oligonucleotides, 10 pmol of oligonucleotide was labeled at the 5' terminus with 0.05 mCi [γ-32P]-ATP using T4 Polynucleotide Kinase (New England Biolabs) and the kinase was heat inactivated for 20' at 80°C. For annealing, 4 pmol of the radiolabeled strand was mixed with 6 pmol of unlabeled complementary strand, NaCl was added to a final concentration of 200 mM, and the mixture was heated to 90°C and then cooled slowly (> 60 min) to 23°C. DNA oligonucleotides used in this study are listed in ***SI Appendix* Table I**.

The substrates in **Fig. 1*A,B*** were made by mixing radiolabeled "FD3" and unlabeled "FD2" with unlabeled "FD1". For lanes 7-12 "FD1-MeP" was used in place of FD1; for lanes 13-18, "FD2-MeP" was used instead of FD2.

The T-DNA substrate for **Fig. 1*C,D*** was made by annealing unlabeled strands "T30A" and "T30B" to radiolabeled cross-bar strand "T30C", producing a T-DNA with a 3' ssDNA of 30 dT, a duplex of 35bp, and two non-homologous arms of 30bp each. For lanes 1-6 "T30A MP" was used in place of T30A; for lanes 13-18, "T30B MP" was used instead of T30B.

For **Figs. 2*C*, 3, and 4**, the ars1 origin mimic DNA with two 3' ssDNA tails was made by annealing unlabeled "Ori Bottom 3' tail" to radiolabeled "Ori Top 3' tail". The alternate origin substrates in **Fig. 4** were made by annealing unlabeled "Ars304 Bottom 3' tail" to radiolabeled "Ars304 Top 3' tail" (**Fig. 4*A***, lanes 6-10) and unlabeled "40GC Bottom 3' tail" to radiolabeled "40GC Top 3' tail" (**Fig. 4*B***, lanes 6-10).

DNA unwinding assays: Unless otherwise noted, all reactions (55 µl) were performed at 30 °C and contained 40 nM CMG (as undecamer) and 0.5 nM radiolabeled DNA substrate in a buffer consisting of 20 mM Tris Acetate pH 7.6, 5 mM DTT, 0.1 mM EDTA, 10 mM $MgSO_4$, 50 mM KCl, and 40 µg/ml BSA. CMG was pre-incubated at 30 °C with the DNA substrate for 10' in the presence of 0.2 mM AMP-PNP and the reactions were started by the addition of 5 mM ATP along with 20 nM unlabeled trap DNA to prevent re-annealing of the unwound product. Concentrations and times of addition of other proteins in the reactions are indicated in the figure legends.

After starting the reaction with ATP, reaction aliquots (10 µl) were stopped at the times indicated in the figures by addition of 4 µl of buffer containing 150 mM EDTA/7% SDS. 1 µl Proteinase K was added and the mixture was incubated 10' at 30° C after which 3 µl STOP/LOAD buffer (0.1M EDTA, 5% SDS, 25% glycerol, and 0.01% each of xylene cyanol and bromophenol blue) was added and the sample was flash frozen in liquid nitrogen. Upon completion of the experiment, flash frozen reaction products were thawed quickly at 30 °C and separated on 15% (**Fig. 1*A***) or 10% (**all other Figures**) native PAGE mini gels by electrophoresis at 100V in TBE buffer. Gels were washed in distilled water, mounted on Whatman 3MM paper, wrapped in plastic and exposed to a phosphor screen that was scanned on a Typhoon 9400 laser imager (GE Healthcare). Scanned gels were analyzed using ImageQuant TL v2005 software.

The trap oligos for the experiment in **Fig. 1*B*** are unlabeled "FD1" and "FD3comp", which binds to unwound FD3 and prevents it from reannealing. The trap oligo for the experiment in **Fig. 1*D*** is unlabeled "T30C". The trap oligo for the ars1 duplex substrates in **Figs. 2-4** is "Ori Bottom 5' tail", which binds to the unwound radiolabeled DNA, forming a forked structure that shifts it to a unique position in the native PAGE gel. For the experiments in **Figs 4*A* and 4*B*, lanes 6-10**, the shift-trap oligos are "Ars304 Bottom 5' tail" and "40GC Bottom 5' tail", respectively. Oligo sequences are shown in ***SI Appendix*** **Table I**.

EMSA assays: The "Ori Bottom 3' tail" oligo (***SI Appendix*** **Table I**) was radiolabeled and used in two separate annealing reactions with either "Ori Top no tail" or "Ori Top 3' tail" oligos to yield the 1-tailed DNA (a single 3' tail) and the origin mimic 2-tailed DNA (two 3' tails), respectively, in **Fig. 1*A***. Both substrates were subsequently PAGE-purified. Binding reactions were performed by incubating 0.5 nM ^{32}P-DNA with increasing amounts of CMG (as indicated) in 10 μL reactions containing 20 mM Tris-acetate, 8% glycerol, 0.02 mM EDTA, 10 mM Na-acetate 10 mM $MgSO_4$ and 0.2 mM AMP-PNP. Reactions were incubated 60 min at 30°C, then directly loaded on a 4% native PAGE gel in TBE buffer containing 5 mM $MgSO_4$. Electrophoresis was performed at 4°C at 240 V for 45 min in TBE buffer supplemented with 5 mM $MgSO_4$. Gels were wrapped in plastic and exposed to a phosphor screen that was scanned on a Typhoon 9400 laser imager (GE Healthcare).

SUPPLEMENTARY FIGURES

SI Appendix Figure S1.

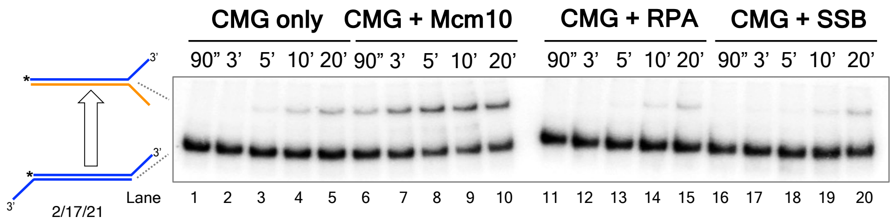

SI appendix,* Figure S1. Other DNA binding proteins do not substitute for Mcm10 in promoting duplex unwinding.** The reaction in **Fig. 2*C was repeated using yeast RPA (lanes 11-15) or *E. coli* SSB (lanes 16-20) in place of Mcm10. Control reactions showing CMG only (lanes 1-5) and CMG + Mcm10 (lanes 6-10) were run side-by-side with the RPA and SSB reactions.

SI Appendix Figure S2.

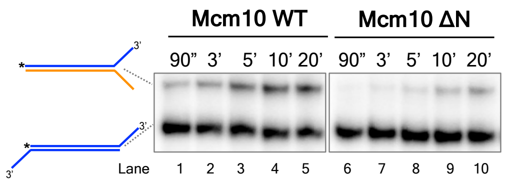

SI appendix,* Figure S2. An N-terminally truncated Mcm10 does not substitute for Mcm10 in promoting duplex unwinding.** The reaction in **Fig. 2*C was repeated using an N-terminally truncated form of Mcm10 (Mcm10ΔN = Mcm10$^{129\text{-}571}$, lanes 6-10) and compared with the WT Mcm10 control (lanes 1-5). CMG is present in all lanes.

SI Appendix Figure S3.

Original Ars1 150mer Sequence (28.7% G/C)

GAAATAGGTTATTACTGAGTAGTATTTATTTAAGTATTGTTTGTGCAC
TTGCCTGCAGGCCTTTTGAAAAGCAAGCATAAAAGATC**TAAACATAAA**
ATCTGTAAAATAACAAGATGTAAAGATAATGCTAAATCATTTGGCTTT
TTGATT

Ars1 150-mer sequence modified to 40% G/C (changes distributed throughout sequence, avoiding ARS consensus sequence and the ends)

GAAATAGGTTAGTACTGAGTCGTATCTATGTAAGTCTTGTGTGTGCAC
TTGCCTGCAGGCCTGTTGAAGAGCCAGCATAACAGATC**TAAACATAAA**
ATCTGTAAAAGAACACGATGTGAAGACAATGCGACATCATGTGGCTTT
TTGATT

***SI appendix,* Figure S3. Sequence of Ars1 150mer modified to 40% G/C.** The ARS1/ARS416 sequence used in **Figs. 2, 3 and 4A** is shown at the top with the ARS consensus sequence highlighted in bold blue. The modified sequence is shown below with the altered bases shown in red.

SI Appendix Table I

**Oligo Name**	**DNA Oligo Sequence (5'-3)**	**Modifications**
FD1	GATCCTGTAATGTCCTAGCAAGCCAGAATTCGGCAGCGTCGCGATCTGCAGCCTTGCCAGAAATCTAGTGTTTTTTTTTTTTTT*T*T*T*T*T*T	* = phosphorothiorate linkages between the bases
FD2	CACTAGATTTCTGGCAAGGCTGCAGATCGCGACGCTGCCG	
FD3	TTTTTTTTTTTTTTTTTTTTTTTTTTTTTTTTTTTTTTTTTAATTCTGGCTTGCTAGGACATTACAGGATC	
FD3comp	GATCCTGTAATGTCCTAGCAAGCCAGAATT	
FD1-MeP	GATCCTGTAATGTCCTAGCAAGCCAGAATTmpCmpGmpGmpCmpAmpGmpCmpGmpTmpCmpGmpCmpGmpAmpTmpCmpTmpGmpCmpAGCCTTGCCAGAAATCTAGTGTTTTTTTTTTTTTT*T*T*T*T*T	Methylphosphonate linkages between bases are indicated by mp<br>* = phosphorothiorate linkages between the bases
FD2-MeP	CACTAGATTTCTGGCAAGGCmpTmpGmpCmpAmpGmpAmpTmpCmpGmpCmpGmpAmpCmpGmpCmpTmpGmpCmpCmpG	Methylphosphonate linkages between bases are indicated by mp
T30A	5'_AGACTGCCATACCCTCACACACCACCGCTATGTAATGTCCTAGCAAGCCAGAATTCGGCAGCGTCTTTTTTTTTTTTTTTTTTTTTTT*T*T*T*T*T*T*T_3'	Phosphorothioate linkages between bases are indicated by *
T30A MP	5'_AGACTGCCATACCCTCACACACCACCGCTATmpGmpTmpAmpAmpTmpGmpTmpCmpCmpTmpAmpGmpCmpAmpAmpGmpCmpCmpAmpGAATTCGGCAGCGTCTTTTTTTTTTTTTTTTTTTTTTTT*T*T*T*T*T*T_3'	Methylphosphonate linkages between bases are indicated by mp
T30B	5'_GACGCTGCCGAATTCTGGCTTGCTAGGACATTACAGGAATTATACTGTCACCAACCACGAGATTT_3'	

T30B MP	5'_GACGCTGCCGAATTCTmpGmpGmpCmpTmpTmpGmpCmpT<br>mpAmpGmpGmpAmpCmpAmpTmpTmpAmpCmpAmpGGAAT<br>TATACTGTCACCAACCACGAGATTT_3'	Methylphosphonate linkages between bases are indicated by mp
T30C	5'_AAATCTCGTGGTTGGTGACAGTATAATTCCTAGCGGTGGTGT<br>GTGAGGGTATGGCAGTCT _3'	
Ori Top 3' tail	GAAATAGGTTATTACTGAGTAGTATTTATTTAAGTATTGTTTGT<br>GCACTTGCCTGCAGGCCTTTTGAAAAGCAAGCATAAAAGATCTA<br>AACATAAAATCTGTAAAATAACAAGATGTAAAGATAATGCTAAA<br>TCATTTGGCTTTTTGATTTTTTTTTTTTTTTTTTTTTTTTTTTTTT<br>TTTTTTT*T*T*T*T*T*T	* = phosphorothiorate linkages between the bases
Ori Top no tail	GAAATAGGTTATTACTGAGTAGTATTTATTTAAGTATTGTTTGT<br>GCACTTGCCTGCAGGCCTTTTGAAAAGCAAGCATAAAAGATCTA<br>AACATAAAATCTGTAAAATAACAAGATGTAAAGATAATGCTAAA<br>TCATTTGGCTTTTTGATT	
Ori Bottom 3' tail	AATCAAAAAGCCAAATGATTTAGCATTATCTTTACATCTTGTTA<br>TTTTACAGATTTTATGTTTAGATCTTTTATGCTTGCTTTTCAAA<br>AGGCCTGCAGGCAAGTGCACAAACAATACTTAAATAAATACTAC<br>TCAGTAATAACCTATTTCTTTTTTTTTTTTTTTTTTTTTTTTTT<br>TTTTTTT*T*T*T*T*T*T	* = phosphorothiorate linkages between the bases
Ori Bottom 5' tail	TTTTTTTTTTTTTTTTTTTTTTTTTTTTTTAATCAAAAAGCCAA<br>ATGATTTAGCATTATCTTTACATCTTGTTATTTTACAGATTTTA<br>TGTTTAGATCTTTTATGCTTGCTTTTCAAAAGGCCTGCAGGCAA<br>GTGCACAAACAATACTTAAATAAATACTACTCAGTAATAACCTA<br>TTTC	
40GC Top 3' tail	GAAATAGGTTAGTACTGAGTCGTATCTATGTAAGTCTTGTGTGT<br>GCACTTGCCTGCAGGCCTGTTGAAGAGCCAGCATAACAGATCTA<br>AACATAAAATCTGTAAAAGAACACGATGTGAAGACAATGCGACA<br>TCATGTGGCTTTTTGATTTTTTTTTTTTTTTTTTTTTTTTTTT*T*<br>T*T*T*T*T	* = phosphorothiorate linkages between the bases
40GC Bottom 3' tail	AATCAAAAAGCCACATGATGTCGCATTGTCTTCACATCGTGTTC<br>TTTTACAGATTTTATGTTTAGATCTGTTATGCTGGCTCTTCAAC<br>AGGCCTGCAGGCAAGTGCACACACAAGACTTACATAGATACGAC<br>TCAGTACTAACCTATTTCTTTTTTTTTTTTTTTTTTTTTTT*T*<br>T*T*T*T*T	* = phosphorothiorate linkages between the bases
40GC Bottom 5' tail	TTTTTTTTTTTTTTTTTTTTTTTTTTTTTTAATCAAAAAGCCAC<br>ATGATGTCGCATTGTCTTCACATCGTGTTCTTTTACAGATTTTA<br>TGTTTAGATCTGTTATGCTGGCTCTTCAACAGGCCTGCAGGCAA<br>GTGCACACACAAGACTTACATAGATACGACTCAGTACTAACCTA<br>TTTC	
Ars304 Top 3' tail	GATGAGATAATATTGTCTATTATATTGCCAATAACAACCAGCGC<br>CAGGTGTACTAAGTCGAGATGACAGAAAATTTATAATAAGTGTA<br>AATACAATAAATTTTGTAAATAAATAACTACTTCAATAGAAATC<br>TCAAAGTCGAAGAAACACTTTTTTTTTTTTTTTTTTTTTTT*T*<br>T*T*T*T*T	* = phosphorothiorate linkages between the bases
Ars304 Bottom 3' tail	GTGTTTCTTCGACTTTGAGATTTCTATTGAAGTAGTTATTTATT<br>TACAAAATTTATTGTATTTACACTTATTATAAATTTTCTGTCAT<br>CTCGACTTAGTACACCTGGCGCTGGTTGTTATTGGCAATATAAT<br>AGACAATATTATCTCATCTTTTTTTTTTTTTTTTTTTTTTT*T*<br>T*T*T*T*T	* = phosphorothiorate linkages between the bases
Ars304 Bottom 5' tail	TTTTTTTTTTTTTTTTTTTTTTTTTTTTTTGTGTTTCTTCGACTT<br>TGAGATTTCTATTGAAGTAGTTATTTATTTACAAAATTTATTGT<br>ATTTACACTTATTATAAATTTTCTGTCATCTCGACTTAGTACACC	

	TGGCGCTGGTTGTTATTGGCAATATAATAGACAATATTATCTCATC	

References for Supplementary Material

1. L. A. Henricksen, C. B. Umbricht, M. S. Wold, Recombinant replication protein A: expression, complex formation, and functional characterization. *J Biol Chem* **269**, 11121-11132 (1994).

2. N. Yao, J. Hurwitz, M. O'Donnell, Dynamics of beta and proliferating cell nuclear antigen sliding clamps in traversing DNA secondary structure. *J Biol Chem* **275**, 1421-1432 (2000).
